# Retrieval of broken bone biopsy needle from the sacroiliac joint – A case report and review of literature

**DOI:** 10.1016/j.tcr.2020.100395

**Published:** 2021-01-02

**Authors:** Viraj N. Gandbhir, Kumar Dussa, Ghanshyam Kakadiya, Nischay K.K., Aseem Parekh

**Affiliations:** Department of Orthopaedics, T.N.M.C. and B.Y.L. Nair Ch. Hospital, Mumbai 400008, Maharashtra, India

**Keywords:** Broken needle, Bone biopsy, Needle removal, Needle in a joint, Drill bit, Needle fracture

## Abstract

Bone biopsies whether Computed Tomography guided or open, are one of the commonest procedures undertaken. Our literature review proves that bone biopsy needle fracture in a bone is a rare complication with no literature available on a needle fracture in a joint. We report a 7-year-old male who underwent an open needle biopsy. During the procedure, the bone biopsy needle fractured with the distal 2.7 cm fragment being completely embedded in the right sacroiliac joint. Considering the location of the fragment, the standard techniques described in literature for extraction could not be applied due to intra-articular nature of the fragment and the risk of complications. We describe a method using a 2.5 mm drill bit to safely extract the foreign body. We have found that reasonable erosion of adjacent cortex, exposes the needle tip, prevents the needle from shattering and avoids further articular damage. There was an uneventful 15 months follow up. This case highlights the fact that bone biopsy procedure mandates correct technique and supervision and as far as possible a disposable pre-sterilized bone biopsy needle should be used.

## Introduction

Trephine biopsy is a well-established and common procedure with limited risks and complications. Trephine needle biopsy breakage in the bone is very rare [1]. Only a handful of cases and the extraction techniques have been documented in literature [2,3]. Furthermore, extraction of the broken fragment can be extremely challenging and if not done carefully can have disastrous consequences. In the case of our 7-year old male patient, as the needle was completely inside the bone and traversing a joint, traditional methods described could not be applied in our case. We discuss our experience with the use of a 2.5 mm drill bit in extracting the completely hidden needle fragment. We have also explored the possible reasons for needle breakage with literature support.

## Case report

**Fig. 1:**
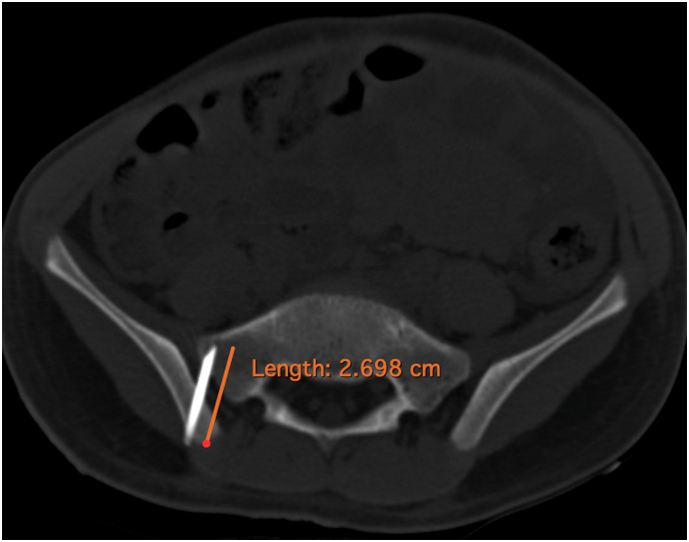
CT scan image of the foreign body measuring about 2.7 cm in length penetrating the right sacroiliac joint.

A 7-year-old male child was admitted in the Paediatric department of our tertiary care hospital for evaluation of eosinophilia. A bone marrow biopsy from posterior iliac crest was planned for definitive diagnosis. After sedation and left lateral position, an autoclaved reusable 14-gauge trephine biopsy needle of unknown make was inserted percutaneously after all aseptic precautions. The inner stylet was removed and the hollow needle was advanced. Due to the resistance felt, the operating surgeon tilted the needle to change the trajectory while the needle was still in the bone. This led to a needle facture with the distal part being embedded in the bone. The procedure was aborted and the broken needle fragment was left in situ. Subsequently an Orthopedic Department consultation was made and X-rays along with a Computed Tomography (CT) scan was ordered to locate the broken needle. The radiological investigations showed the presence of 2.7 cm needle fragment traversing the right sacroiliac joint with the needle tip lying just 3 mm posterior to the anterior cortex of the sacrum (Fig. 1). As the needle was lying in the joint, after counselling the parents, it was decided to extract the needle fragment.

**Fig. 2:**
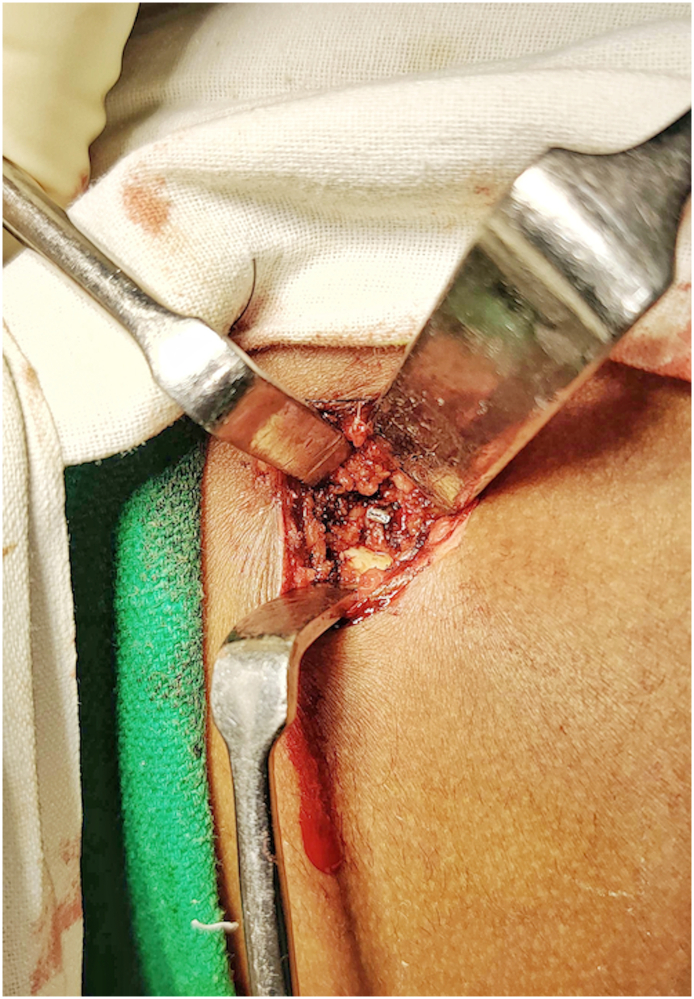
Visualisation of needle end after drilling the surrounding cortex.

**Fig. 3:**
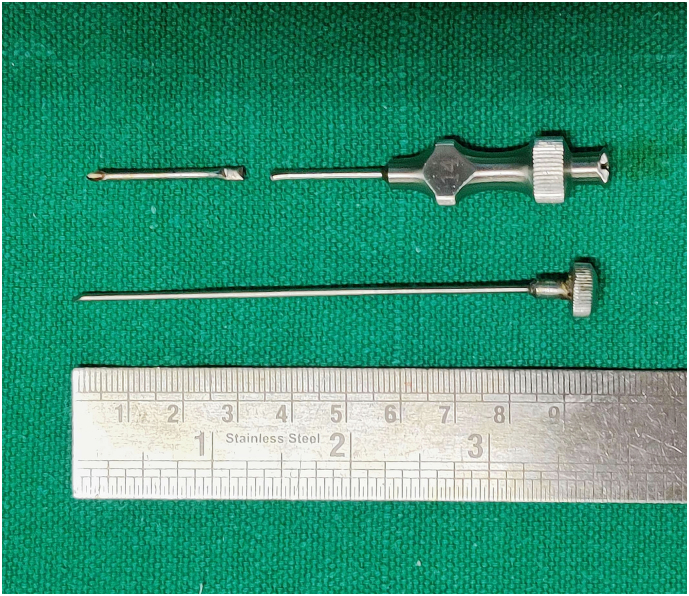
Biopsy needle after retrieval.

In the operating room, after induction with general anaesthesia, preoperative antibiotics were administered and the patient was placed in left lateral position. As there were two puncture marks 2 cm apart over the skin, it was decided to visualize the foreign body under fluoroscopy to aid in localization. A skin incision of 2.5 cm was made and after superficial and deep dissection the needle end was localized which was bent and buried in the cortex. The surrounding cortex had to be undermined using a drill bit of 2.5 mm to expose the needle end which was then removed with a plier (Fig. 2). Confirmatory C arm shoots were taken. The needle length was measured and was found to be matching with the CT scan measurement (Fig. 3). The core of bone extracted from the broken needle was placed in formalin and sent for histopathological analysis. Post thorough wash the wound was instilled with a local anaesthetic agent and closed in layers. A dressing was applied, and the patient was extubated uneventfully. The patient was allowed to bear weight as tolerated following the procedure as the defect was not deemed a significant fracture risk. The patient had an uneventful 15 month follow up.

## Literature review

A literature search was performed with the PubMed search engine of the National Library of Medicine of the Institutes of Health (http://www.ncbi.nlm.nih.gov/pubmed) using following combinations of keywords: “Trephine biopsy needle;” or “Bone biopsy needle;” and “Broken;” “Fracture;” “Extraction”. The search was restricted to English-language publications with no date limitations (Table 1). A descriptive summary is included under Discussion.

**Table 1:** Summary of cases with broken bone biopsy needle in the bone published in English.

Authors	Age/sex	Indication for bone biopsy	Type of bone biopsy	Type of bone biopsy needle used	Location of the broken needle	Joint involvement?	Needle tip outside the bone?	Broken needle extracted?	Method of extraction
Asprey et al. [3]	15 years/female	Osteoid osteoma of Proximal tibia	CT guided	14-gauge Bonopty	Left proximal tibia	No	No	Yes	Cannulated with K-wire and extracted with a cannulated reamer
Shaikh et al. [2]	43 years/male	Sclerotic metastatic lesion of T 11 vertebral body	CT guided	15-guage Bonopty	T 11 vertebra	No	No	Yes	11 guage Murphy M2 needle advanced over it and broken needle extracted
Huang et al. [5]	NA	NA	NA	NA	Proximal Femur	No	Yes	Yes	NA
Bain et al. [1]	NA	NA	NA	NA	Iliac Crest	No	NA	Yes	NA
	NA	NA	NA	NA	Iliac Crest	No	NA	No	NA
Maciel et al. [6]	NA	NA	NA	NA	NA	No	NA	Yes	NA
Our case	7 years/male	Definitive diagnosis of eosinophilia	Open biopsy	Autoclaved reusable biopsy needle	Right sacroiliac joint	Yes	No	Yes	Erosion of cortex with a drill bit to expose the needle and later extracted.

NA: Not Available; CT: Computed Tomography.

## Discussion

Trephine biopsy procedures whether CT-guided or open, are generally considered to be safe [1]. Complications as a result of the procedure lead to increased cost, duration of stay, physical and mental agony to the patient and sometimes even death of patients [1]. Fortunately, device failure as a complication is rare. It is so rare that out of the two year review (2002 and 2003) and 26,653 cases analyzed by Bain et al. [1,4] through the British Society of Haematology, only two instances of trephine biopsy needle fracture were reported. It is possible that such device failures are considered as relatively minor complications compared to infection and hemorrhage and hence are under-reported.

Our extensive literature search revealed only six cases with bone biopsy needle fracture as a documented complication (Table 1) [[1], [2], [3],^5^,^6^]. In all the cases, the broken fragment was lying in the bone without involvement of the adjacent joint. The broken needle was extracted in five cases and left in situ in one patient. In two patients, the broken needle fragment was completely hidden inside the bone. It had to be extracted by a larger cannulated drill bit in one patient and by using a larger Murphy M2 needle (Cook Medical, Bloomington, Illinois, United States) in the other. The information on the type of bone biopsy needle used is available in two patients and in both cases Bonopty set (AprioMed, Uppsala, Sweden) was used.

The correct technique of harvesting bone marrow from the posterior iliac crest includes passage of the biopsy needle parallel to the iliac crest [7]. In our case, looking at the trajectory of the needle, it is possible that the parallelism was not maintained enhancing the fact that even a small degree of angulation may cause it to penetrate the posterior sacroiliac joint and other vital structures around the area [7]. Though other complications like neurovascular injury, pseudoaneurysm formation and gluteal compartment syndrome due to incorrect angulation have been noted in literature before [8,9], penetration of the posterior sacroiliac joint has never been documented as far as we can tell. It is possible that the posterior sacroiliac joint may have been breached unintentionally during a number of procedures but fortunately the needle was never fractured. The hard feel during needle insertion and inability to aspirate the marrow in such cases, is sufficient for the surgeon to correct the needle course.

A presterilized disposable needle must always be preferred over an autoclavable and reusable one to prevent transmission of diseases and avoid the risk associated with cleaning the needle [10]. Moreover, repeated sterilization cycles as is normally done with reusable needles, have been shown to affect the hardness of the metal and even causing corrosion in some cases [11]. Due to cost constraints and unavailability of disposable needle in our set up, a reusable stainless steel needle was used. We believe this coupled with erroneous technique could have led to the catastrophic device failure.

As in our case, as the broken needle was traversing the sacroiliac joint, a decision was made to remove the needle. A broken biopsy needle fragment penetrating and occupying a joint and its extraction has never been reported before in literature to the best of our knowledge.

A number of methods of extraction of broken needles in the bone have been documented [2,3]. Needle in needle technique using a K-wire [2] or a broken screw removal set could not be used in our case as there was a possibility of damaging the articular surface of the sacroiliac joint with a larger cannulated drill bit or a hollow reamer. Further, as the needle tip was lying just 3 mm from the anterior sacral cortex, it was felt that usage of a hollow reamer could have easily perforated the anterior cortex thereby putting vital anatomical structures at risk. As demanded by needle-in-needle technique [2], a K-wire for percutaneous retrieval could also not be introduced as the needle was completely buried under the cortex. In our case, careful drilling of adjacent bone prior to extraction, exposed the needle tip without compromising the structural integrity of the bone or the joint. Care was taken to make sure that the rotating drill bit does not touch the foreign body. Once the surrounding cortex was sufficiently undermined, using narrow tip pliers, the tip of the needle was firmly grasped. The needle was then successfully removed by rotating and pulling, taking care not to bend the needle. A 2.5 mm or an appropriate sized drill bit is freely available in most orthopedic operating rooms and a diamond burr may also be used as an alternative. In our opinion, this simple precautionary measure greatly aided in the success of the procedure without jeopardizing the safety and can be easily reproduced.

More information is needed regarding the brands of bone biopsy needle commonly used and regarding the needles that fractured. Due to cost restraints, a reusable autoclaved needle, which has more chances of breakage was used in our patient. Our case highlights the extraction technique and the fact that wherever possible a pre-sterilized disposable needle should be used which minimizes the chance of breakage.

## Statement of informed consent

The patient and his legal guardians were informed that data concerning the case would be submitted for publication and they agreed.

## Declaration of competing interest

The case report was not presented at any meeting wholly or partly and there is no conflict of interest.
